# Tempol relieves lung injury in a rat model of chronic intermittent hypoxia via suppression of inflammation and oxidative stress

**DOI:** 10.22038/ijbms.2018.31716.7714

**Published:** 2018-12

**Authors:** Yeying Wang, Bing Hai, Li Ai, Yu Cao, Ran Li, Hui Li, Yongxia Li

**Affiliations:** 1 Department of Respiratory Medicine, The Second Affiliated Hospital of Kunming Medical University, Kunming, Yunnan 650101, People’s Republic of China; 2 Department of Epidemiology and Biostatistics, School of Public Health, Kunming Medical University, Kunming, Yunnan 650500, People’s Republic of China

**Keywords:** Inflammation response, Intermittent hypoxia, NF-κB, Nrf2/HO-1, Oxidative stress, Tempol

## Abstract

**Objective(s):**

Obstructive sleep apnea (OSA) is confirmed to cause lesions in multiple organs, especially in the lung tissue. Tempol is an antioxidant that has been reported to restrain inflammation and oxidative stress, with its role in OSA-induced lung injury being unclear. This study aimed to investigate the beneficial effect of tempol on chronic intermittent hypoxia (IH)-induced lung injury.

**Materials and Methods:**

A rat model of OSA was established by IH. There were four groups: normal air (NA), IH, IH+tempol, NA+tempol. Inflammatory response was evaluated by TNF-α, IL-1β, and IL-6 levels. Oxidative stress was detected by MDA and GSH levels, and SOD activity. The protein levels were assessed by Western blot. DNA binding activity of NF-κB or Nrf2 was determined by electrophoretic mobility shift assay.

**Results:**

According to the results, tempol administration alleviated pathological changes of the lung tissue, decreased leukocyte count and protein content (*P<*0.001) in bronchoalveolar lavage fluid (BALF). Inflammation response in lung tissue induced by IH was suppressed by tempol as evidenced by decreased levels of TNF-α, IL-1β, and IL-6 (*P<*0.001) and protein levels of COX-2 and iNOS (*P<*0.001). Moreover, tempol inhibited oxidative stress in lung tissue by down-regulating the MDA level (*P<*0.001) and enhancing SOD activity (*P<*0.001) and the GSH level (*P<*0.05). In addition, tempol repressed inflammation response via inactivation of the NF-κB pathway. Furthermore, the results suggested that tempol repressed oxidative stress by activating the Nrf2/HO-1 pathway.

**Conclusion:**

Our findings suggest that tempol effectively relieves OSA-induced lung injury.

## Introduction

Obstructive sleep apnea syndrome (OSAS) as a common chronic sleep disorder with 2%-7% incidence in adults worldwide, is characterized by recurrent apnea, heavy snoring, and daytime sleepiness (1). In recent years, growing evidence has demonstrated that OSAS may lead to multiple organ lesions, including lung tissue, in chronic intermittent hypoxia (IH) animal models. Lung tissue is uniquely vulnerable to IH, and its pathophysiologic manifestations are alveolar hypoventilation and pulmonary artery vasoconstriction (2). In addition, IH may result in severe inflammatory response in the lung tissue and eventually promote the formation of pulmonary fibrosis (3). A previous study also suggested that oxidative stress damage occurred in the process of IH-induced lung injury (4). Therefore, the effective suppression of inflammation and oxidative stress may be a potential therapeutic strategy for IH-induced lung injury.

Tempol is a superoxide dismutase mimetic agent and exhibits extensive antioxidant activities by scavenging reactive oxygen species (ROS). It has been shown that tempol administration could restrain IH-induced inflammation in heart tissue (5). Tempol was suggested to alleviate respiratory muscle dysfunction in IH-treated rats and may be an adjunct treatment for OSAS (6). In addition, tempol repressed sleep apnea-induced hypertension via decreasing ET-1 production in rats (7). Previous research indicated that atherosclerosis could be caused by IH, while tempol significantly inhibited endothelial cell apoptosis via attenuating oxidative and inflammatory injury (8). Based on the above data, we expect that tempol may protect against lung injury induced by IH through diminishing inflammation and oxidative stress. 

In this study, we investigated the protective role of tempol in lung injury induced by IH and elucidated the potential molecular mechanisms in a rat model of OSAS. 

## Materials and Methods

***Animal model***

Healthy male Wistar rats (200-220 g) were purchased from HFK Bioscience Co., LTD (Beijing, China) and maintained in standard conditions with free access to food and water. The rats were randomly divided into four groups (n=6 per group): normal air (NA) group, intermittent hypoxic (IH) group, IH+tempol group, and NA+tempol group. The rats in IH and IH+tempol groups were placed in a chamber and subjected to 5% O_2 _(60 sec)/21% O_2_ (60 sec) cycles between 9:00 am and 5:00 pm every day for 12 consecutive weeks. While the rats in NA and NA+tempol groups received normal air containing 21% O_2_. Tempol was dissolved in freshly prepared drinking water (1 mM) every day and administered to rats, during the 12-week NA or IH treatment. The dosage of tempol was determined according to previous studies (7, 9). Whereas the rats of other groups were given regular water without tempol. The rats were anesthetized by intraperitoneally injecting with 50 mg/kg pentobarbital sodium 12 weeks after the IH treatment. Peripheral blood and the bronchoalveolar lavage fluid (BALF) of the rats were collected. Then the rats were sacrificed and the lung tissues were obtained for further experiments. All animal experimental procedures were approved by the Institutional Animal Care and Use Committees of The Second Affiliated Hospital of Kunming Medical University.

***HE staining***

The rat lung tissues were fixed in 4% paraformaldehyde overnight and embedded in paraffin. Then the samples were cut into 5-μm sections and subjected to routine HE staining. The images were taken at 200 × magnification by a light microscope (Olympus, Japan).

***Bradford assay***

The protein concentration in BALF was assessed by Bradford Protein Assay Kit (Beyotime, China) according to the instructions. The protein concentration was calculated according to the bovine serum albumin standard curves.

***Giemsa staining***

The cell count of total, mononuclear, and multinuclear leukocytes was determined by Giemsa staining. In brief, 10 μl of BALF suspension was dropped onto a glass slide and incubated with Giemsa (Nanjing Jiancheng Bioengineering Institute, China) following the instructions. Under a microscope, the leukocytes were classified and counted.

***ELISA***

The levels of TNF-α, IL-1β, and IL-6 in lung tissues were evaluated by commercially-available ELISA kits (Boster, China). The HO-1 level was detected by an ELISA Kit for Heme Oxygenase 1 (Cloud-Clone Corp. USA). The results were calculated according to the standard curves using standard proteins.

***Western blot analysis***

Western blot was performed as previously described (10). Briefly, frozen lung tissues of rats were homogenized in RIPA Lysis Buffer (Beyotime) containing 1% PMSF (Beyotime) and kept on ice for 5 min. After centrifugation, the protein samples in supernatants were denatured and the concentration was assessed by an Enhanced BCA Protein Assay Kit (Beyotime). Equal quantity (40 µg) of protein was subjected to sodium dodecyl sulfate-polyacrylamide gel electrophoresis (SDS-PAGE) and then transferred to polyvinylidene fluoride (PVDF) membranes (Millipore, USA). Whereafter the membranes were blocked with 5% skimmed milk for 1 hr and incubated with corresponding primary antibodies against COX2 (1:700, Proteintech, USA), iNOS (1:7500, Proteintech, USA), IκBα (1:1000, Cell Signaling Technology, USA), p- IκBα (1:1000, Cell Signaling Technology, USA), NF-κB (1:1000, Proteintech, USA), HO-1 (1:500, Boster, China), Nrf2 (1:500 Boster, China), GAPDH (1:5000, Bioss, China), and Histone H3 (1:1000, Proteintech, USA) at 4 ^°^C overnight. Subsequently, HRP-conjugated goat anti-rabbit secondary antibody (Beyotime) was added. Then, the results were visualized with a BeyoECL Plus (Beyotime).

***Immunohistochemistry***

Immunohistochemistry was performed according to a previous study (11). The lung tissues were embedded in paraffin, cut into 5-μm sections and transferred onto glass slides. Immunohistochemical staining was carried out using primary antibodies against COX-2 (1:50, Proteintech, USA) and iNOS (1:50, Proteintech, USA) at 4 ^°^C overnight, respectively. Then, the slides were stained with Biotin-labeled Goat Anti-Rabbit IgG(H+L) (1:200, Beyotime, China) at 37 ^°^C for 30 min, followed by incubation with HRP-labeled Streptavidin (1:200, Beyotime) at 37 ^°^C for 30 min. After being stained with DAB (Solarbio, China) and counterstained with hematoxylin (Solarbio, China), the slides were observed and photographed under a light microscope at a magnification of 400 ×.

***Detection of MDA, GSH levels, and SOD activity***

The lung tissues were homogenized in cold PBS, received three repeated freeze-thaw cycles, and centrifuged. MDA, GSH levels, and SOD activity were assessed by commercially available kits purchased from Jiancheng Bioengineering Institute (Nanjing, China), following the manufacturer’s instructions. 

***Electrophoretic mobility shift assay (EMSA)***

EMSA was carried out as previously described (12). The DNA binding activities of NF-κB and Nrf2 in lung tissues were assessed by EMSA using commercial kits (Viagene, China) following the manufacturer’s protocol. Briefly, the nuclear proteins were isolated from lung tissues by a Nuclear and Cytoplasmic Protein Extraction Kit (Beyotime, China). Then, the nuclear protein (25 µg) was incubated with the biotin end-labeled probe (0.5 µl) in binding buffer for 20 min at room temperature. The reaction mixtures were subjected to electrophoresis with a 6.5% polyacrylamide gel at 180 V for 80 min, blotted onto a nylon membrane, cross-linked, and visualized by chemiluminescence.

***Statistical analysis***

All results were presented as mean± standard deviation (SD). The statistical analyses were carried out using GraphPad Prism 5 software. One-way ANOVA followed by Bonferroni’s Multiple Comparison Test was used to determine the significant differences among multiple experimental groups. Statistical significance was set at *P*<0.05.

## Results

***Effect of tempol on IH-induced lung injury***

**Figure 1:**
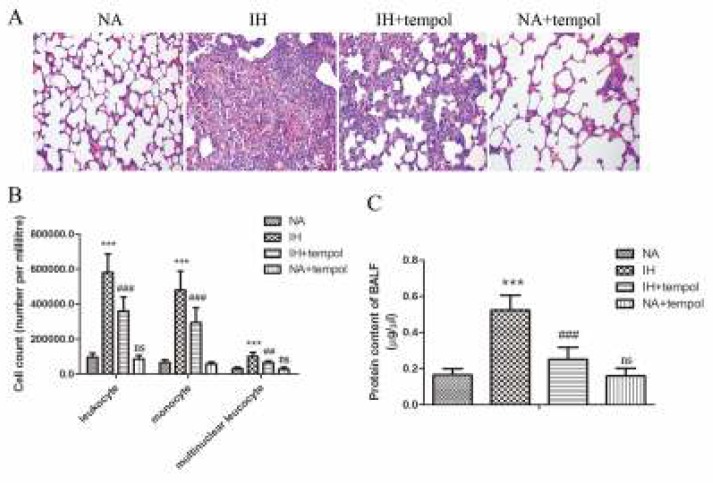
Tempol alleviated intermittent hypoxia-induced lung injury. (A) The pathomorphological changes of lung tissues were observed by HE staining. (B) The numbers of leukocytes, monocytes, and multinuclear leukocytes in BALF were detected. (C) The protein content in BALF was determined. The experimental data were expressed as mean±SD (n=6). *** *P<*0.001, versus the NA group. ##* P<*0.01, ### *P<*0.001, versus the IH group. ns, no significance, versus the NA group

The pathological changes in lung tissue were observed by HE staining and shown in Figure 1(A). Compared with the NA group, IH treatment led to broken alveoli and increased infiltration of inflammatory cells in the pulmonary tissues, whereas administration of tempol could effectively alleviate these pathological changes. As assayed by Giemsa staining, the number of total, mononuclear, and multinuclear leukocytes in BALF was distinctly raised by IH treatment (*P<*0.001), which could be notably reduced by tempol (Figure 1(B)) (for leukocyte and monocyte, *P<*0.001; for multinuclear leukocyte, *P<*0.01). Moreover, Figure 1(C) revealed that IH induced a marked increase in the protein content of BALF (*P<*0.001). However, tempol treatment could significantly reduce the protein content (*P<*0.001). There was no significant difference between NA and NA+tempol groups (*P>*0.05). 

***Effect of tempol on the IH-induced inflammatory response in lung tissue***

**Figure 2:**
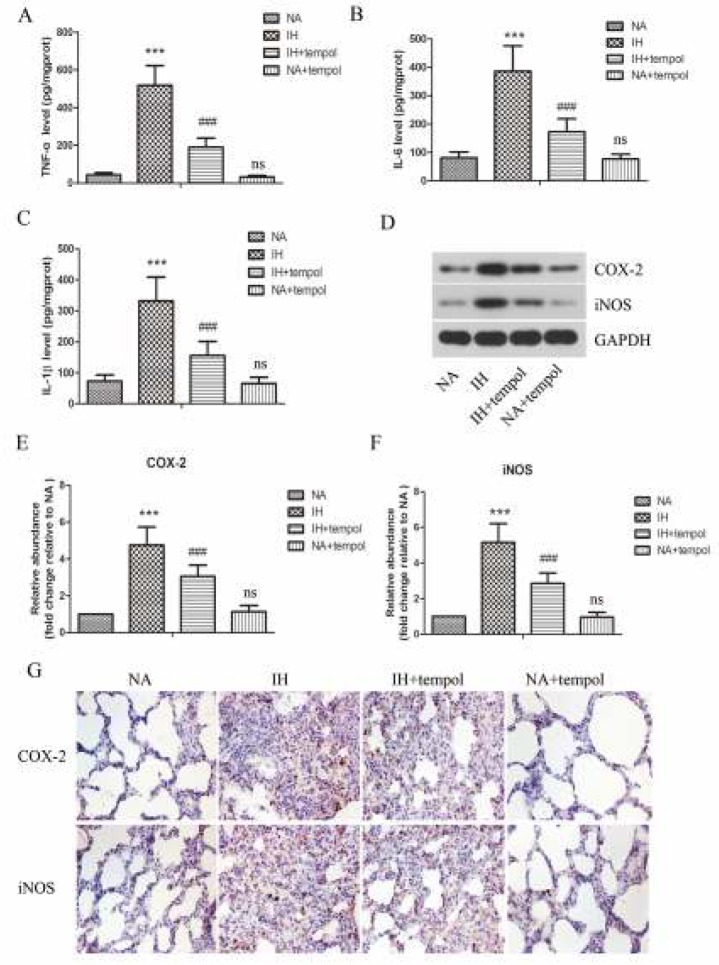
Tempol restrained intermittent hypoxia-induced inflammatory response in lung tissues. The levels of TNF-α (A), IL-6 (B), and IL-1β (C) in lung tissues were assessed by ELISA. (D) The protein levels of COX-2 and iNOS were determined by Western blot assay. GAPDH was used as a loading control. (E-F) The gray-scale value of the bands was quantitatively analyzed. (G) The expressions of COX-2 and iNOS in lung tissues were evaluated by immunohistochemical staining. The experimental data were expressed as mean±SD (n=6). *** *P<*0.001, versus the NA group. ### *P<*0.001, versus the IH group. ns, no significance, versus the NA group

The levels of pro-inflammatory cytokines, including TNF-α, IL-1β, and IL-6 in lung tissues were assessed by ELISA. As illustrated in Figure 2 (A-C), the levels of TNF-α, IL-1β, and IL-6 were obviously up-regulated by IH as compared with NA (*P<*0.001), which were strikingly restrained by tempol administration (*P<*0.001). As COX-2 and iNOS play critical roles in inflammatory response, we further determined their levels in lung tissues. As detected by Western blot and shown in Figure 2 (D-F), treatment with tempol distinctly repressed IH-induced increased protein levels of COX-2 and iNOS (*P<*0.001). The results showed no significant difference between NA and NA+tempol groups (*P>*0.05)**. **Furthermore, the protein expression of COX-2 and iNOS in lung tissue was evaluated by immunohistochemical staining. As presented in Figure 2G, the results further confirmed that IH-induced enhanced expression of COX-2 and iNOS was suppressed by tempol administration. 

***Effect of tempol on IH-induced oxidative stress in lung tissue***

**Figure 3:**
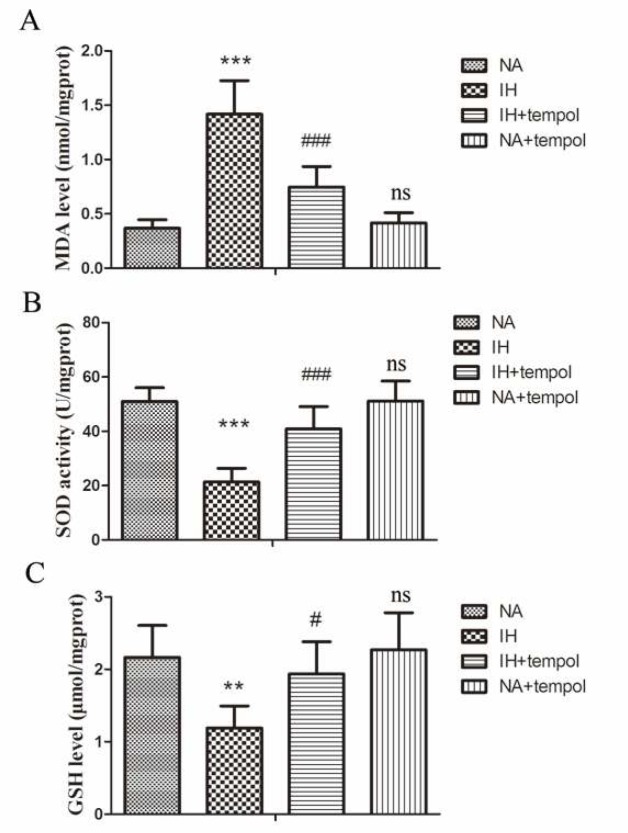
Tempol repressed intermittent hypoxia-induced oxidative stress in lung tissues. The level of MDA (A), activity of SOD (B), and GSH level (C) were assessed by commercial kits. The experimental data were expressed as mean±SD (n=6). ** *P<*0.01, *** *P<*0.001, versus the NA group. # *P<*0.05, ### *P<*0.001, versus the IH group. ns, no significance, versus the NA group

To evaluate oxidative stress injury, the levels of MDA, GSH, and activity of SOD in lung tissue were assessed. As presented in Figures 3 (A-C), IH resulted in an obvious increase in MDA level (*P<*0.001), while decreasing SOD activity (*P<*0.001) and GSH level (*P<*0.01) in lung tissue. However, administration of tempol could reverse the above changes induced by IH (for MDA and SOD, *P<*0.001; for GSH, *P<*0.05). We found no significant difference between NA and NA+tempol groups (*P>*0.05). These results illustrated that tempol treatment significantly relieved IH-induced oxidative stress injury in lung tissue.

***Effect of tempol on IH-induced activation of the NF-*** ***κB*** ***signaling pathway***

**Figure 4:**
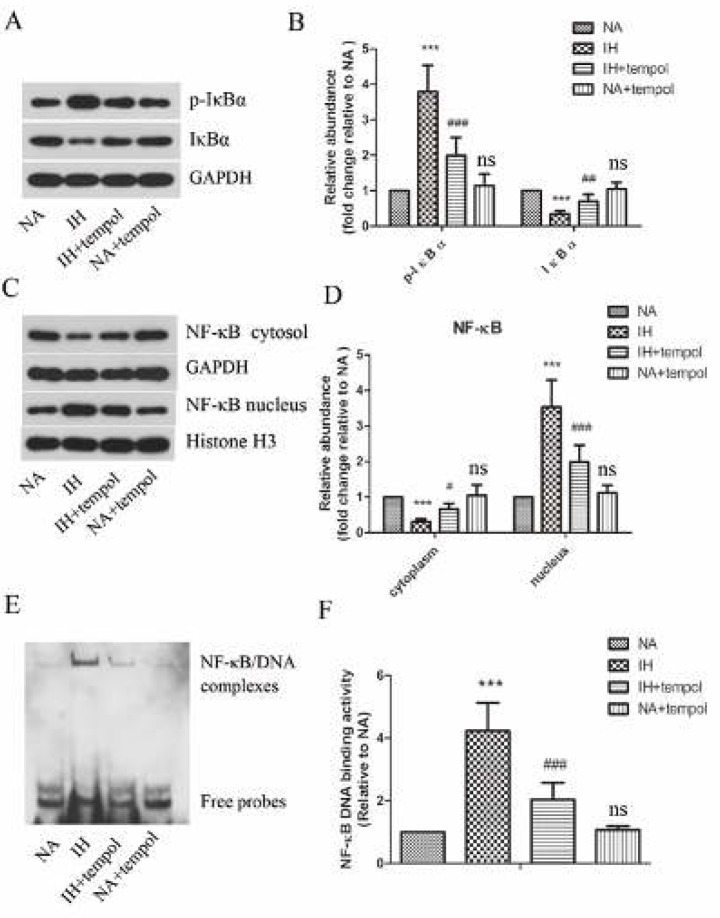
Tempol inhibited intermittent hypoxia-induced activation of NF-κ B signaling pathway. (A)&(C) The protein levels of p-I κ B α, I κ B α, cytoplasmic NF-κ B, and nuclear NF-κ B were determined by Western blot assay. GAPDH was used as a loading control. (B)&(D) The gray-scale value of the bands was quantitatively analyzed. (E) DNA binding activity of NF-κ B in lung tissues was assessed by EMSA. (F) Quantitative analysis of NF-κ B DNA binding activity. The experimental data were expressed as mean ± SD (n=6). *** *P<*0.001, versus the NA group. # *P<*0.05, ## *P<*0.01, ### *P<*0.001, versus the IH group. ns, no significance, versus the NA group

As stated above, tempol had a protective effect against inflammatory response and oxidative stress induced by IH. To further elucidate the potential mechanism, we focused on the NF-κ B signaling pathway. As shown in Figures 4 (A-D), the protein levels of p-IκBα and nuclear NF-κB were enhanced, while the levels of IκBα and cytoplasmic NF-κB dramatically declined in the IH treatment group (*P<*0.001 for all). However, tempol effectively restrained these IH-induced changes (for p-IκBα and nuclear NF-κB, *P<*0.001; for IκBα, *P<*0.01; for cytoplasmic NF-κB, *P<*0.05). In addition, tempol administration remarkably suppressed IH-induced enhanced DNA binding activity of NF-κB in lung tissue (Figures 4 E&F) (*P<*0.001 for all). There was no significant difference between NA and NA+tempol groups (*P>*0.05). These observations show that the NF-κB signaling pathway was activated by IH, which was obviously restrained by tempol administration. 

***Effect of tempol on the activation of the Nrf2/HO-1 signaling pathway***

**Figure 5:**
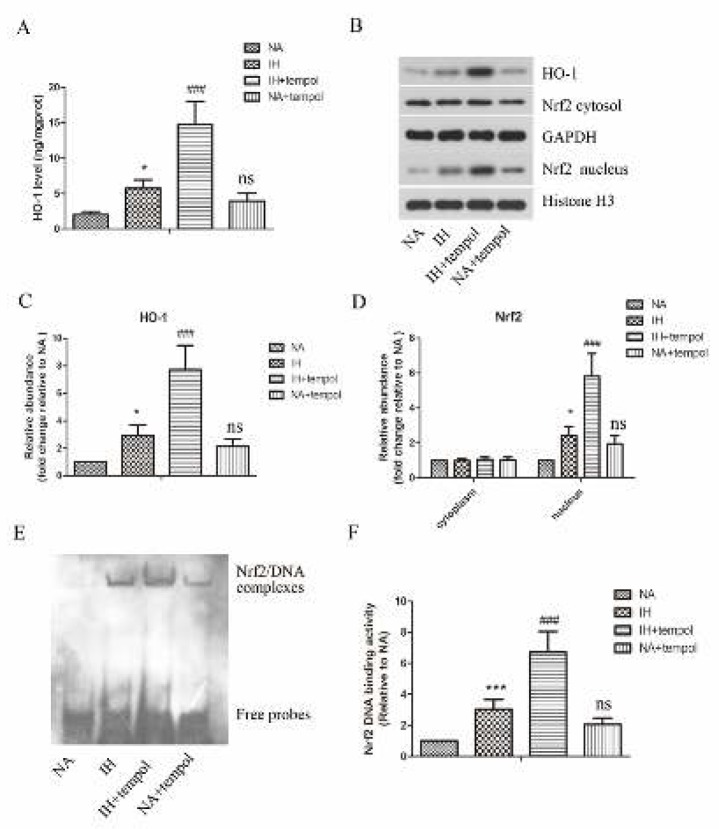
Tempol further promoted intermittent hypoxia-induced activation of the Nrf2/HO-1 signaling pathway. (A) The level of HO-1 in lung tissues was detected by a commercial kit. (B) The protein levels of HO-1, cytoplasmic Nrf2, and nuclear Nrf2 in lung tissues were measured by Western blot assay. GAPDH was used as a loading control. (C)& (D) The gray-scale value of the bands was quantitatively analyzed. (E) DNA binding activity of Nrf2 in lung tissues was determined by EMSA. (F) Quantitative analysis of Nrf2 DNA binding activity. The experimental data were expressed as mean±SD (n=6). * *P<*0.05, *** P<0.001, versus the NA group. ### *P<*0.001, versus the IH group. ns, no significance, versus the NA group

Nrf2/HO-1 signaling pathway has been confirmed to play crucial roles in counteracting oxidative stress damage. Therefore, we also investigated the effect of tempol on the activation of the Nrf2/HO-1 signaling pathway. As illustrated in Figure 5A, tempol treatment further promoted IH-induced up-regulation of HO-1 level in lung tissue (*P<*0.001). Moreover, as assayed by Western blot and shown in Figures 5(B-D), the protein levels of HO-1 and nuclear Nrf2 in lung tissue were raised by IH (*P<*0.05 for all), which could be further enhanced by tempol treatment (*P<*0.001 for all). There was no significant difference in cytoplasmic Nrf2 level among the different treatment groups (*P>*0.05 for all). Furthermore, the DNA binding activity of Nrf2 was strengthened by IH (*P<*0.001), which could be further boosted by tempol treatment (Figures 5 E &F) (P<0.001). The results showed no significant difference between NA and NA+tempol groups (*P>*0.05). These findings indicated that tempol facilitated IH-induced activation of the Nrf2/HO-1 signaling pathway.

## Discussion

It has been widely acknowledged that lung injury, as a serious complication, can occur in OSAS. In this study, we aimed to investigate the protective effect of tempol on lung injury in a rat model of IH. The results suggested that tempol treatment effectively ameliorated IH-induced lung injury, inflammatory response, oxidative stress injury via regulating NF-κB, and HO-1/Nrf2 signaling pathways. 

Tempol is a membrane-permeable radical scavenger and has been demonstrated to protect against lung injury in a variety of other models. For example, evident protection by tempol against lipopolysaccharide-induced acute lung injury was reported by El-Sayed *et al.* *(*13*)*. A previous study reported also illustrated that tempol could reduce remote lung injury caused by intestinal ischemia/reperfusion (14). It was found that tempol attenuated zymosan-induced multiple organ failure, including lung injury in rats (15). Based on these studies, tempol is very likely to alleviate IH-induced lung injury. According to our results, IH-induced pathological changes of lung tissue, increased leukocyte number, and protein content in BALF were restrained by tempol administration, indicating the effectiveness of tempol treatment.

The inflammatory response that was regulated by multiple pro-inflammatory cytokines plays pivotal roles in the progression of IH-induced lung injury. An example of this is the research performed by our lab, in which IH resulted in obvious inflammatory response as evidenced by increased expressions of TNF-α, IL-1β, and IL-6 in lung tissues (16). The excessive expressions of these pro-inflammatory cytokines may further exacerbate lung injury and lead to lung edema and alveolar hemorrhage (17). TNF-α is an important pro-inflammatory cytokine and upregulated in OSAS patients, which has a close correlation with the severity of OSAS (18). IL-6 is also a pro-inflammatory cytokine that participates in the recruitment of chemokines and leukocytes, and the level of IL-6 is elevated in patients with OSAS (19, 20). IL-1β released by macrophages may promote inflammatory cell activation and thus strengthen inflammatory response (21). In this study, administration with tempol significantly suppressed IH-induced excessive expressions of TNF-α, IL-1β, and IL-6 in lung tissues. Moreover, TNF-α, IL-1β, and IL-6 can stimulate the expression of iNOS that may enhance the level of NO, a pro-inflammatory mediator (22, 23). COX-2 is a key enzyme that catalyzes arachidonic acid into prostaglandins and participates in inflammatory response (24). The levels of iNOS and COX-2 were found to be increased by IH in our previous study. In the present study, tempol treatment could significantly restrain the IH-induced increase in iNOS and COX-2 levels. There, results indicated that inhibiting inflammatory response was involved in the beneficial effect of tempol against IH-induced lung injury.

Growing evidence has demonstrated that oxidative stress takes part in various tissue injuries associated with IH (25, 26). MDA is the product of lipid peroxidation and its level positively correlated with the degree of oxidative stress injury. A rat model of IH was established by Zhao* et al*, in which the MDA level was obviously enhanced by IH (27). Oxidative stress may occur when there is an imbalance between oxidation and anti-oxidation (28). A series of enzymes, such as SOD, and non-enzymatic antioxidants including GSH may compose the anti-oxidization system in the body (29). SOD and GSH play pivotal roles in neutralizing ROS (29). Our results showed that IH-induced decrease in MDA level and increase in SOD activity and GSH level were effectively suppressed by tempol, indicating that tempol alleviated oxidative stress damage in IH-induced lung injury. 

To evaluate the potential mechanisms of tempol in the treatment of IH-induced lung injury, we focused on NF-κB and Nrf2/HO-1 signaling pathways. NF-κB is an important transcription factor that participates in the regulation of various biological processes via binding to its target genes. The activation of NF-κB can be motivated by pro-inflammatory cytokines, such as TNF-α (30). Moreover, NF-κB amplifies the inflammatory response, particularly through promoting the release of pro-inflammatory cytokines (31). However, under normal condition, NF-κB is inactivated in the cytoplasm via binding to its inhibitory protein IκBα. The phosphorylation of IκBα can be enhanced by stimuli, then IκBα is degraded, which promotes the nuclear translocation of NF-κB. According to our results, tempol treatment reduced p- IκBα level, raised IκBα level, and restrained IH-induced activation of the NF-κB signaling pathway. 

Nrf2 is a transcription factor that plays crucial roles in oxidative stress (32). Nrf2 is anchored in the cytoplasm under normal conditions, whereas under oxidative stress state, it may translocate into the nucleus to facilitate the expressions of anti-oxidative molecules. HO-1 is a well-confirmed anti-oxidative gene, which is downstream of Nrf2 (33). The activation of the Nrf2/HO-1 pathway has been suggested to protect against IH-induced oxidative stress injury (34). Our results demonstrated that the Nrf2/HO-1 pathway was significantly activated by IH, which was further enhanced by tempol treatment. Thus, tempol relieved IH-induced oxidative stress in the lung tissue via activating the Nrf2/HO-1 pathway. 

## Conclusion

Taken together, tempol treatment alleviated IH-induce lung injury via inhibiting inflammatory response and oxidative stress. The inhibition of NF-κB and promotion of HO-1/Nrf2 signaling pathways were involved in the protective mechanisms of tempol. This study may provide evidence for tempol as a potential drug for the treatment of lung injury in OSAS.
